# Sigma-1 Receptor-Modulated Neuroinflammation in Neurological Diseases

**DOI:** 10.3389/fncel.2018.00314

**Published:** 2018-09-20

**Authors:** Jia Jia, Jian Cheng, Cheng Wang, Xuechu Zhen

**Affiliations:** ^1^Jiangsu Key Laboratory of Neuropsychiatric Diseases and College of Pharmaceutical Sciences, Soochow University, Suzhou, China; ^2^Institute of Neuroscience, Soochow University, Suzhou, China; ^3^Department of Pharmacy, Suzhou Science and Technology Town Hospital, Suzhou, China

**Keywords:** sigma-1 receptor, neuroinflammation, neurological diseases, microglia, astrocytes

## Abstract

A large body of evidence indicates that sigma-1 receptors (Sig-1R) are important drug targets for a number of neuropsychiatric disorders. Sig-1Rs are enriched in central nervous system (CNS). In addition to neurons, both cerebral microglia and astrocytes express Sig-1Rs. Activation of Sig-1Rs is known to elicit potent neuroprotective effects and promote neuronal survival via multiple mechanisms, including promoting mitochondrial functions, decreasing oxidative stress and regulating neuroimmnological functions. In this review article, we focus on the emerging role of Sig-1Rs in regulating neuroinflammation and discuss the recent advances on the Sig-1R-modulating neuroinflammation in the pathophysiology and therapy of neurodegenerative disorders.

## Introduction

The sigma-1 receptors (Sig-1Rs) are endoplasmic reticulum (ER) transmembrane proteins. They exert chaperoning functions and modulate physio-pathological processes in the central nervous system (CNS). As chaperone proteins, Sig-1Rs do not exhibit biological functions independent of other protein partners. Sig-1Rs are expressed widely in brain cells, including neurons, astrocytes, microglia and oligodendrocytes (Gundlach et al., 1986; Alonso et al., 2000; Hayashi and Su, 2003a,b, 2004; Gekker et al., 2006; Ruscher et al., 2011; Zhao et al., 2014). Numerous studies show that Sig-1Rs promoted neuronal survival and restored neuronal functions in neurodegenerative diseases via multiple mechanisms, including the modulation of calcium homeostasis and glutamate activities, attenuation of reactive species production, modulation of ER and mitochondrial functions, regulation of reactive gliosis and neuronal plasticity (Nguyen et al., 2015, 2017; Ruscher and Wieloch, 2015). In addition to Sig-1R, sigma receptor sigma-2 (Sig-2R) has also been shown to play an important role in neurological diseases (detailed to see the review from Guo and Zhen, 2015).

Neuroinflammation refers to the inflammatory responses mediated by microglial, astroglial and endothelial cells in CNS. During neuroinflammation, microglia-mediated inflammatory responses display dual effects depending on the activating signals (Hanisch and Kettenmann, 2007; Xiong et al., 2016). In one hand, microglial activation in response to tissue damage or dysfunction leads to phagocytosis and the release of pro-inflammatory mediators. On the other hand, microglia may promote an anti-inflammatory and regenerative milieu depending on their polarization state (Patel et al., 2013). For instance, microglia undergo prominent morphological changes along with various functional states, displaying either pro-inflammatory or anti-inflammatory functions following cerebral ischemia. Therefore, activated microglia can be either neurotoxic or neuroprotective to CNS. A large body of studies suggests that targeting neuroinflammation represents a therapeutic intervention for treating neurodegenerative diseases. The fact that Sig-1Rs are expressed in glial cells suggests Sig-1Rs as potential targets in treating CNS diseases with neuroinflammation dysfunction. In this review article, we review current findings about the effects of Sig-1Rs on neuroinflammation in the major neurodegenerative diseases.

## Sigma-1 Receptors and Astrocytes

Astrocyte activation is considered as a key feature of neuroinflammation. Depending on the insults, reactive astrocytes proliferate and release exocytotic gliotransmitters, pro-inflammatory cytokines as well as neurotropic factors such as BDNF. Sig-1Rs are highly expressed in astrocytes (Ruscher et al., 2011; Francardo et al., 2014). In primary rat astrocytes, pretreatment of the Sig-1R antagonist BD1047 inhibits methamphetamine-mediated upregulation of Sig-1R and GFAP expression (Zhang et al., 2015a). Compared to WT astrocytes, methamphetamine failed to increase the GFAP expression in primary astrocytes isolated from Sig-1R KO mice. Furthermore, in rat C6 glioma cells, a rat cell line of astrocytic origin, methamphetamine induced nuclear factor-κB (NF-κB) p65 translocation into the nucleus. The effect was reversed by BD1047 (Zhang et al., 2015b). NF-κB p65 signaling contributes to diverse pathological processes, including neuroinflammation. In neuropathic mice, blockade of Sig-1Rs by BD1047 prevents mechanical allodynia possibly via inhibiting astrocyte activation induced by the chronic constriction injury. Modulating p-p38 may be the underlying mechanism (Moon et al., 2014). In a mouse model of motor neuron degeneration, chronic treatment of Sig-1R agonist PRE-084 also decreased reactive astrocytosis (Peviani et al., 2014). In neuronal-glial mixed cultures isolated from the Sig-1R KO mice, GFAP expression was enhanced compared to that of WT (Weng et al., 2017). These results indicate that Sig-1Rs modulate neuroinflammation. Meanwhile, in response to inflammatory stress, glial cells may release BDNF to ameliorate injury (Béjot et al., 2011; Zhang et al., 2012; Chen et al., 2015). For instance, a novel Sig-1R agonist stimulated BDNF release from rat primary cortical astrocytes (Malik et al., 2015). Consistently, two metabolites of haloperidol (reduced haloperidol) that act as functionally selective Sig-1R agonists stimulate human astrocytes to secret BDNF. (Dalwadi et al., 2017). In support, we recently found that SKF83959, a recent identified Sig-1R allosteric modulator (Guo et al., 2013), elicited rapidly antidepressant effects via stimulating BDNF production (Wang et al., 2016).

## Sigma-1 Receptor and Microglia

Microglia, the resident macrophages in the brain and spinal cord, are the primary mediator of neuroinflammation and play both beneficial and detrimental roles in neurodegenerative diseases. Accumulating evidence suggests a dual role of microglia in neurodegeneration diseases and recovery, which depends on the functional phenotypes of the microglia (Hu et al., 2015; Xiong et al., 2016; Lan et al., 2017; Ma et al., 2017; Deczkowska et al., 2018). Microglia are typically classified into classic M1 phenotypes and alternative M2 phenotypes although it was challenged recently (Ransohoff, 2016). M1 polarized microglia are traditionally considered as pro-inflammatory and detrimental to the CNS, while M2 polarized microglia are anti-inflammatory and protective to the CNS by promoting neuronal repair and regeneration. Activation of microglial cells contributes to pathological development in many neurological diseases such as stroke, Alzheimer’s disease (AD) and Parkinson’s disease (PD). Inhibition of M1 microglial polarization and promotion of M2 polarization are considered as an important approach for the drug discovery and therapy of neurological and neurodegenerative disorders (Jin et al., 2014; Zhang et al., 2017b). In the peripheral immune system, Sig-1R activation potently suppresses the inflammatory responses induced by a variety of stimuli (Bourrié et al., 2002). The Sig-1R ligands SR31747A and SSR125329A enhanced lipopolysaccharide (LPS)- or Staphylococcal enterotoxin B (SEB)-induced serum release of the anti-inflammatory cytokine interleukin-10 (IL-10), and concomitantly inhibited the production of pro-inflammatory cytokine tumor necrosis factor-α (TNF-α; Bourrie et al., 1995; Bourrié et al., 1996, 2002). In contrast, SR31747A induced a reduction in NO and IL-10 release in LPS-stimulated RAW 264.7 macrophages dose-dependently (Gannon et al., 2001). The contradictory results suggest that peripheral macrophages may not be the responding cells of the increased anti-inflammatory IL-10 production by SR31747A. Sig-1Rs are expressed in microglia (Gekker et al., 2006) and mediate anti-inflammatory effects in CNS. Evidence shows that Sig-1R may regulate microglia polarization. Chao reported that pretreating BV-2 cells with the Sig-1R antagonist BD1047 significantly reduced the methamphetamine-induced increased the ratio of M1 marker (iNOS) and M2 marker (Chao et al., 2017). An *in vitro* study shows that Sig-1Rs stimulation suppressed the morphological, migratory and the inflammatory responses of microglia to LPS (Hall et al., 2009). Afobazole, a putative Sig-1R ligand, suppresses microglial activation and migration in response to ATP and UTP. These effects of afobazole were attenuated by either Sig-1R or Sig-2R antagonists (Cuevas et al., 2011). The strong evidence from treatment of Sig-1R knockout mice with PRE-084 did not elicit anti-neuroinflammatory effect (Francardo et al., 2014). Similarly, using LPS-stimulated murine BV2 microglial cells, our recent report showed that a potent allosteric Sig-1R modulator SKF83959 suppressed the expression of the pro-inflammatory mediators, including TNF-α, interleukin-1β (IL-1β) and inducible nitric oxide synthase (iNOS), the release of NO and the generation of reactive oxygen species (Wu et al., 2015). In particularly, the anti-inflammatory effects of SKF83959 were blocked by selective Sig-1R antagonists (BD1047 or BD1063), indicating a Sig-1R-mediated event. Furthermore, we showed that the suppression of microglia activation by SKF83959 was independent of either MAPK/ERK or IKK/IκB signaling pathways. The finding was not consistent with the report that (+)-pentazocine, a Sig-1R agonist, suppressed the microglia activation via inhibition of LPS-induced MAPK/ERK pathway in retinal microglia (Zhao et al., 2014). Moreover, (+)-pentazocine only at certain doses (1 μM and 10 μM) reduced apoptotic cell death via ERK1/2 pathway in BV2 microglia under hypoxia/reoxygenation conditions (Heiss et al., 2016). This is interesting because it suggests that allosteric modulator of Sig-1R modulate microglia-mediated neuroinflammation via different mechanism from the receptor agonist.

## Sigma-1 Receptors and Stroke

Stroke is a serious cerebrovascular disease that leads to high morbidity and mortality worldwide. Unfortunately, the effective stroke treatments are rather limited. In clinical, thrombolytic therapy with tissue plasminogen activator (tPA) is the only therapeutic intervention for ischemic stroke during acute phase. However, the narrow therapeutic window of tPA limited the clinical application of tPA. Thus, developing effective therapies for stroke is urgent demand. Ischemic stroke, the dominant subtype of stroke, is characteristic of focal cerebral ischemia due to major cerebral arteries occlusion. Reduction of regional cerebral blood supply and lack of oxygen trigger inflammatory responses in consequent with irreversible injury in the brain. Subsequently, reperfusion of brain with delayed restoration of blood and oxygen induces more severe inflammatory responses, which causes secondary injury to the brain. Therefore, modulation of neuroinflammation after stroke is a potential therapeutic intervention for cerebral ischemia. Interestingly, increased Sig-1R expression in the penumbra neuron followed the acute stroke was recently reported (Zhang et al., 2017a).

Evidence suggests that Sig-1R ligands exert neuroprotective effects against cerebral ischemia. Literatures indicate that the neuroprotective effects against stroke conferred by the activation of Sig-Rs can be attributed to the modulation of inflammatory responses. For instance, cerebral ischemia injury strongly enhanced the mRNA level and protein expression of pro-inflammatory cytokines including monocyte chemoattractant protein-1 (MCP-1) and IL-1β. Treatment with Sig-1R agonist dimemorfan (at the onset of reperfusion) significantly reduced the mRNA and protein expression of MCP-1 and IL-1β, in accordance with inhibition of the NF-κB signaling pathway (Shen et al., 2008). Consistently, in the *in vivo* LPS-induced endotoxin shock model, treatment with dimemorfan significantly decreased plasma TNF-α concentrations, neutrophil infiltration and oxidative stress induced by LPS in mice. These results suggest that the anti-inflammatory action of dimemorfan contributes to the protective effects of dimemorfan against LPS-induced endotoxin shock in mice (Wang et al., 2008). Recently, it was shown that treatment with Sig-1R agonist PRE-084 at 3 h and 24 h after the embolic stroke onset significantly reduced infarct volumes and improved neurological deficits by inhibiting pro-inflammatory cytokines and enhancing anti-inflammatory cytokines, such as IL-10 and IL-4 following cerebral ischemia (Allahtavakoli and Jarrott, 2011). In agreement with this observation, Sig-1R activation affects Iba1 expression in microglia/macrophages of the ischemic hemisphere after experimental stroke (Ruscher et al., 2012).

Interestingly, a recent report showing that administration of a highly selective Sig-1R antagonist, SIRA (E-52862/MR309) intracerebroventricularly or intravenously in the focal cerebral ischemia model also significantly reduced the infarct sizes and neurological deficits in mice following middle cerebral artery occlusion (MCAO). The underlying mechanism of the neuroprotective effects exerted by SIRA may be associated with the significant reduction in metalloproteinase-9 (MMP-9) expression, astrogliosis and microglial proliferation (Sánchez-Blázquez et al., 2018). It will be very important to further study how and why Sig-1R agonists and antagonists produced the similar neuroprotective or neurorestorative effects in stroke.

## Sigma-1 Receptors and Traumatic Brain Injury (TBI)

Similar to stroke, acute brain injury following trauma can also lead to long-term neurological deficits. Recently, a study used the controlled cortical impact (CCI) model and demonstrated that the Sig-1R selective agonist PRE-084 played a beneficial role in traumatic brain injury (TBI) by reducing microglia activation, nitrosative and oxidative stress to proteins (Dong et al., 2016). In this report, they administered PRE-084 systemically to mice 15 min after TBI injury and then assessed brain edema and behavior biochemical measurements in animals following 28 days after TBI. PRE-084 significantly decreased brain edema and attenuated neurologic deficits. One of the mechanisms underlying the neuroprotective effects of PRE-084 against TBI may be the suppression of microglia activation as indicated by decreased Iba1 immunoreactivity. However, further investigation is needed to examine whether the concomitant application of Sig-1 R antagonists block the neuroprotective effects of PRE-084 and distinguish polarization status of the microglia/macrophages.

## Sigma-1 Receptors and Parkinson’s Disease

The neuroprotective and neurorestorative effects of the Sig-1R agonists have also been reported in experimental model of PD. The important functional roles of Sig-1R in PD has been reported before (Mishina et al., 2005; Paquette et al., 2009; Mori et al., 2012; Hong et al., 2015). A recent study reported the potential antiparkinsonian effect of Sig-1R agonist. Chronic treatment of PRE-084 improves motor impairment in the 6-hydroxydopamine (6-OHDA)-lesioned mouse PD model. A significant reduction of the number of CD68-positive microglia and macrophages was detected in PRE-084-treated mice (Francardo et al., 2014). These finding suggest that Sig-1R activation-induced reduction in neuroinflammation may account for the functional recovery in PD model. In addition, the potential beneficial effects of Sig-1R ligands in levodopa-induced dyskinesia in PD patient and experimental animals have been also reported (Paquette et al., 2009; Fox et al., 2017). This data indicated that modulation of Sig-1R may be a potential new drug target for PD therapy.

## Sigma-1 Receptors and Amyotrophic Lateral Sclerosis

Amyotrophic Lateral Sclerosis (ALS) is a fatal neurodegenerative disease which is characterized by progressive loss of motor neurons in the spinal cord and brain (Boillée et al., 2006). Intracellular accumulations of mutant, misfolded proteins are major pathological hallmarks of ALS and related disorders. A landmark discovery in 1993 reported that the mutations in superoxide dismutase 1 (SOD1) account for 20% of the inherited ALS cases (Rosen et al., 1993). ALS is also recognized as a non-cell autonomous disease. The motor neurons expressing mutant SOD1 induce the initial disease onset and early progression but do not contribute to later disease progression. Interestingly, microglia or astrocytes expressing mutant SOD1 exacerbate disease progression after onset without influence on initial timing of disease onset (Ilieva et al., 2009).

In the CNS, Sig-1Rs are highly expressed in motor neurons (Mavlyutov et al., 2010). ALS-causative mutation (E102Q) of Sig-1R has been found in juvenile ALS (Al-Saif et al., 2011). Expression of mutant sig-1R-E102Q in neuro2A cells reduces mitochondrial ATP production, disrupts mitochondrial structure and aggravates ER stress-induced neuronal death (Fukunaga et al., 2015). Since mitochondrial dependent signaling controls innate and adaptive immune responses, mitochondrial dysfunction in glial cells likely at least in part regulates neuroinflammation. Sig-1R accumulations were also observed in enlarged C-terminals and ER structures of alpha motor neurons of ALS patients and also in SOD1 transgenic mice (Prause et al., 2013). Knockout the Sig-1R in the SOD1*G93A mouse ALS model reduces the longevity of the mice, suggesting the lack of Sig-1R exacerbates the pathological progression of ALS (Mavlyutov et al., 2013). Meanwhile, administration of the Sig-1R agonist PRE-084 was shown to slow the progression of ALS in SOD1*G93A mouse ALS model and significantly reduced microglial marker Iba1-1 immunoreactivity in the spinal cord, but did not affect astroglial reactivity (Mancuso et al., 2012). Moreover, co-administration with the Sig-1R antagonist BD1036 reverse the reduction in microglial reactivity induced by PRE-084 in the SOD1 mice. In another study using the wobbler mouse model of motor neuron disease, chronic treatment with PRE-084 displayed beneficial effects on motor performance and improved motor neuron survival, suggesting that PRE-084 may not only confer the neuroprotective effects against SOD1 mutation (Peviani et al., 2014). This observes were also supported with other studies (Ono et al., 2014; Tagashira et al., 2014), indicating that Sig-1R function is not only involved in the pathological development but also a promising drug target for the ALS treatment. Moreover, chronic treatment with PRE-084 SOD1 mutation mice increased the expression of another macrophage/microglial marker CD11b. Especially, the numbers of cells positive for M2 phenotype marker CD206 significantly increased in the white matter of PRE-084-treated mice, in concomitant with the increased microglia and macrophage marker CD68 immunoreactivity (Peviani et al., 2014). This indicated that regulation of the microglia/macrophage polarization may be involved in the neurorestorative effect of Sig-1R activation in ALS. Indicating modulation of microglia activation may also contribute to the beneficial effects of Sig-1R agonist in the treatment of ALS.

## Sigma-1 Receptors and Other Neurodegenerative Diseases

In addition, Sig-1Rs have also been widely indicated in AD, multiple sclerosis (MS) and Huntington diseases. Recently, a novel selective allosteric sigma-1 receptor agonist AF710B was reported to revert the cognitive deficits possibly via neuroinflammation suppression in APP transgenic AD rats (Hall et al., 2018). An earlier *in vitro* study also demonstrated Afobazole, a mixed Sig-1R/Sig-2R agonist, decreased microglial activation in rat primary microglia exposed to Aβ_25–35_ (Behensky et al., 2013). Experimental autoimmune encephalomyelitis (EAE) model of MS is characterized with inflammation and demyelination in the CNS. Oxombre et al. (2015) show that a novel Sig-1R agonist confers protection by decreasing the magnitude of inflammation in EAE.

## Sigma-1 Receptors in Human Brain

Using *in situ* hybridization, Kitaichi et al. (2000) reports that the Sig-1R genes express in the mouse, guinea pig and human brain, with the distribution mainly in hippocampus, hypothalamus and most cortical areas. Since 1996, Sig-1R have been cloned in human and several other species (Hanner et al., 1996; Kekuda et al., 1996; Pan et al., 1998; Seth et al., 1998). Correlation between increased risk of diseases and Sig-1R gene mutation polymorphism has also been reported. For example, TT-P gene mutation of Sig-1R is found to be a risk factor against AD (Fehér et al., 2012). However, to confirm the result, a larger sample size of clinical study is necessary. To date, a few Sig-1R agonists (SA4503 and ANAVEX2-73) have entered clinical trials of neurodegenerative disorders (Lahmy et al., 2013; Urfer et al., 2014). All those efforts may provide a potential new avenue for neurodegenerative disease treatment.

## Conclusion

**Figure 1:**
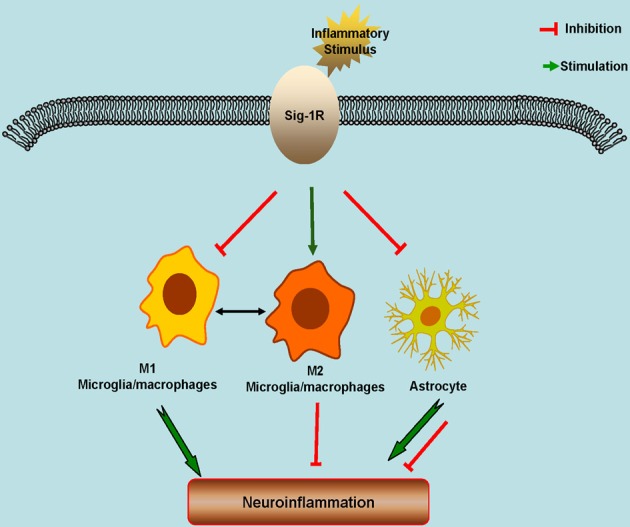
Schematic model showing the potential roles of Sigma-1 Receptors (Sig-1Rs) in neuroinflammation. Sig-1Rs are expressed widely in brain cells, including neurons, astrocytes, microglia and oligodendrocytes. Sig-1R activation promotes the M2 microglial repair/regenerative phenotype, and inhibits the M1 microglial phenotype and the astrocytic response to inflammatory stimuli.

Sig-1R is widely expressed in CNS. Altered Sig-1R expression and functions are associated with the pathophysiology of a number of neurological diseases. Sig-1R plays an essential role in glial functional regulation. Numerous studies have been shown that Sig-1R has a profound effect on neuroinflammation and consequently neuroprotection (Figure 1). As a result, Sig-1R may be a potential drug target particularly for the neuroinflammation-associated diseases such as ALS, stroke, PD and AD. For the future perspectives, development of specific and high selective Sig-1R or Sig-2R ligands is needed. Meanwhile, considering the complex of Sig-1R or Sig-2R functions and enrichment in distribution, allosteric modulator of Sig-1R may provide unique advantage in term of the drug development since allosteric modulation is known to have significant better selectivity with less unwanted effects.

## Author Contributions

JJ and JC wrote the manuscript draft. CW and XZ revised and edited the manuscript.

## Conflict of Interest Statement

The authors declare that the research was conducted in the absence of any commercial or financial relationships that could be construed as a potential conflict of interest.
